# Carotid imaging changes and serum IL-1β, sICAM-1, and sVAP-1 levels in benign paroxysmal positional vertigo

**DOI:** 10.1038/s41598-020-78516-7

**Published:** 2020-12-09

**Authors:** Xiaoxu Chen, Huimin Feng, Hongjin Liu, Xianrong Xu, Jianchang Wang, Zhanguo Jin

**Affiliations:** 1 Aerospace Balance Medical Center, Chinese PLA Air Force Medical Center, Beijing, 100142 China; 2 People’s Liberation Army Troops of 95935 Unit, Changchun, 130000 China; 3 Hebei North University, Zhangjiakou, 075000 China; 4 Department of Medical Identification, Chinese PLA Air Force Medical Center, Beijing, 100142 China; 5 Chinese PLA Air Force Medical Center, Beijing, 100142 China; 6 Aviation Physiology Identification and Training Laboratory, Chinese PLA Air Force Medical Center, Beijing, 100142 China

**Keywords:** Immunology, Biomarkers, Molecular medicine

## Abstract

Benign paroxysmal positional vertigo (BPPV) is the most common cause of vertigo. This study was performed to evaluate serum levels of inflammatory factors and changes in B-mode carotid ultrasound findings in patients with BPPV. The study population consisted of 90 BPPV patients and 90 age- and sex-matched controls. ELISA was used to compare the levels of inflammatory factors, such as interleukin-1β (IL-1β), tumor necrosis factor-alpha (TNF-α), soluble intercellular adhesion molecule-1 (sICAM-1), prostaglandin-E2 (PG-E2), and soluble vascular adhesion protein-1 (sVAP-1), between BPPV patients and controls. In addition, the results of ultrasonographic imaging to determine carotid intima-media thickness (C-IMT), carotid atheromatous plaque, and vertebral artery stenosis were also compared between the BPPV and control groups. Serum levels of IL-1β, sICAM-1, and sVAP-1 were significantly higher in BPPV patients than controls (*P* < 0.001, *P* < 0.05, and *P* < 0.001, respectively). C-IMT and vertebral artery stenosis were significantly different in BPPV patients compared to controls (both *P* < 0.05). There were no significant relations between other parameters and BPPV. IL-1β, sICAM-1, and sVAP-1 are potentially associated with the pathogenesis of BPPV, and C-ITM and carotid vertebral stenosis may be useful reference imaging findings for the diagnosis of BPPV.

## Introduction

Benign paroxysmal positional vertigo (BPPV) is a disorder of the inner ear characterized by brief spinning sensations with changes in head position^1^. Although head trauma and otological disorders occasionally cause BPPV, most cases are idiopathic. Hall et al.^2^ described the pathogenesis of BPPV as involving cast-off otolithic debris with endolymph flow entering the semicircular canal and subsequently inducing classified nystagmus with respect to gravity. This definition of BPPV is widely accepted, but the underlying molecular mechanism remains to be elucidated. The inner ear has a blood–labyrinthine barrier and connects to the cervical lymph nodes, and participates in the inflammatory response by producing cytokines in the spiral ligament (SL)^3^. Recent studies regarding the pathogenesis of BPPV have suggested roles of inflammation and relevant inflammatory factors or biomarkers^4,5^. Satoh et al.^6^ performed antigen challenge by injection of keyhole limpet hemocyanin (KLH) into the cochlea of systemically sensitized mice and detected the expression of cytokines and the accumulation of inflammatory cells in the cochlea on immunohistochemical analysis. Inner ear diseases are mediated by inflammation, which disrupts inner ear function and leads to the release of inflammatory factors that can be detected in the peripheral circulation^4^. These inflammatory factors can be used as biomarkers and provide useful information in the study of BPPV.

Güçlütürk et al.^4^ reported that interleukin-1β (IL-1β) and oxidative stress were responsible for the pathogenesis of BPPV. IL-1β is a pleiotropic proinflammatory cytokine that is involved in autoimmune inner ear disease (AIED)^7–9^. Ichimiya et al.^10^ reported that cultured SL fibrocytes secreted chemokines and other mediators after stimulation with tumor necrosis factor-alpha (TNF-α) or IL-1β, and soluble intercellular adhesion molecule (sICAM-1) secretion was elevated after stimulation with TNF-α but was not detected after stimulation with IL-1β. Using a murine model of cochlear immunity, Satoh et al.^6^ reported that the expression of TNF-α amplified the inflammatory response and interacted with IL-1β to cause cochlear disease. One study showed that TNF-α was produced in cells of the stria vascularis and the SL^11^. Elevated serum IL-1β level stimulated the secretion of prostaglandin-E2 (PG-E2)^12^. An animal study showed that PG-E2 agonists increased the production of vascular endothelial growth factor (VEGF), and VEGF and its receptor were present in the cochlea^13^. sICAM-1 has been suggested to be connected with the component expressed on the surface of activated cells, such as endothelial and smooth muscle cells^14^. One study showed that high glucose and inflammatory cytokines stimulated the production of soluble vascular adhesion protein-1 (sVAP-1) in diabetic patients^15^. sVAP-1, which functions as a semicarbazide-sensitive amine oxidase (SSAO), is a soluble adhesion molecule on activity cells surface which involved in vascular endothelial cells and smooth muscle cells^16^. At present, there is a great deal of interest in the roles of inflammatory factors in BPPV.

B-mode carotid ultrasound is routinely performed to determine carotid intima-media thickness (C-IMT), carotid atheromatous plaque, and vertebral artery stenosis in outpatients with vertigo. C-IMT can be used as an indicator for evaluation of coronary microvascular dysfunction to determine the risk of cardiovascular disease^17^, and reflects accelerated atherosclerosis in chronic inflammatory disease^18^. Vertebral artery stenosis has been suggested to be one of the root causes of transient ischemic attacks (TIAs) and ischemic stroke^19^. Carotid atheromatous plaque is a chronic inflammatory disease of the arteries, and always coexists with increased C-IMT^20^.

Based on these considerations, we hypothesized that the pathogenesis of BPPV was related to inflammation-induced inner ear damage. This study was performed to investigate the inflammatory factors and carotid imaging changes associated with the pathogenesis of BPPV.

## Materials and methods

This study was conducted between October 2016 and October 2017 at Aerospace Balance Medical Center, Beijing, China. The clinical protocol was approved by the Ethics Committee of Chinese PLA Air Force Medical Center, Beijing, China, and all procedures were performed in accordance with relevant guidelines and regulations. After receiving a careful explanation of the aims and procedures of the investigation, written informed consent was obtained from all participants recruited to the study, and informed consent was obtained from a parent and/or legal guardian of subjects who are under 18.

### Patients and clinical study

A total of 180 participants aged 13–87 years were included in the study. The study group consisted of 90 patients diagnosed with idiopathic BPPV based on the clinical practice guidelines for BPPV (2017) of the American Academy of Otolaryngology-Head and Neck Surgery Foundation^21^. Positional nystagmus associated with head position change was determined by the SRM-IV BPPV Diagnosis and Treatment System (Siruimei Medical Technology GmbH, Beijing, China). The inclusion criterion for the study group was positional nystagmus recorded by SRM-IV: simulation of traditional Dix-Hallpike and Roll-test, with multiple characteristic features of latency time, displacement, reversibility, transiency, crescendo and decrescendo pattern, interchangeability and fatigability^22^. The exclusion criteria were nystagmus secondary to other diseases of the inner ear, including Ménière’s disease, vestibular neuritis, sudden sensorial deafness, semicircular canal paralysis, etc., nystagmus secondary to other physical factors that may damage the otolith organs, including a history of surgery, head and/or neck trauma, etc., and taking immunosuppressants and anti-inflammatory drugs within the previous 3 months. Patients with hearing loss, otological surgery, neurological diseases, pregnancy, lactation, hypothyroidism, and other inflammatory and infectious conditions were excluded. The control group consisted of 90 age- and sex-matched subjects without any cochleovestibular disorders selected at random from Physical Examination Center, Beijing, China. Both groups consisted of 28 males and 62 females.

Clinical data, including age, height, weight, ongoing health problems, medication history (hypertension, diabetes, and migraine), and the use of drugs were collected. Ultrasonographic analyses of C-IMT, carotid atheromatous plaque, and vertebral artery stenosis were performed using a color Doppler instrument (ADVIA Centaur IU-22; Philips Healthcare GmbH, Best, Netherlands). C-IMT was evaluated by a professional sonographer, and was classified as follows: Normal, ≤ 0.1 cm; Mild, 0.1 – 0.13 cm; and Severe ≥ 0.13 cm.

### Evaluation of serum inflammatory factor levels

Non-fasting blood samples (5 ml) were collected from all subjects with anticoagulant tubes. Collected specimens were centrifuged at 3000×*g* for 5 min, after which serum was removed to 2-ml centrifuge tubes and frozen at − 80 °C until the time of the assay. Plasma levels of IL-1β, TNF-α, ICAM-1, PG-E2, and sVAP-1 were measured using a human-IL-1β enzyme-linked immunosorbent assay (ELISA) kit (ImmunoWay, Plano, TX), human-TNF-α ELISA kit (ImmunoWay), human-sICAM-1 ELISA kit (Invitrogen, Carlsbad, CA), human-PG-E2 ELISA kit (ImmunoWay), and human-sVAP-1 ELISA kit (ImmunoWay) according to the respective manufacturer’s instructions. The optical density at 450 nm (OD_450_) of each well of the ELISA microplates was measured using a microplate reader (iMark; Bio-Rad, Hercules, CA).

### Statistical analysis

SPSS 19.0 (SPSS Inc., Chicago, IL) was used for statistical analysis. All enumeration data are presented as the mean ± standard deviation (SD), while measurement data are shown as percentages. The unpaired samples *t* test was used for within-group analysis of normally distributed enumeration data. The *χ*^2^ test was used for analysis of measurement data. Non-parametric analyses were carried out using Wilcoxon’s signed rank test. In all analyses, *P* < 0.05 was taken to indicate statistical significance.

## Results

The study population consisted of 90 BPPV patients and 90 age- and sex-matched control subjects. Both groups consisted of 28 males and 62 females. The patients in the BPPV group were aged 13–87 years (mean 55.22 ± 14.67 years). In the BPPV group, 67 (74.4%) patients showed involvement of the posterior semicircular canal, 27 (30%) showed involvement of the horizontal semicircular canal, three (3.3%) showed involvement of the anterior semicircular canal, and seven (7.8%) showed multiple semicircular canal involvement. According to the BPPV classification, the BPPV group consisted of 61 cases (67.8%) of cupulolithiasis and 29 cases (33.2%) of canalithiasis. The current episode of BPPV was not the first episode in 55 cases (61.1%). Table 1 shows the clinical features and carotid imaging examination indexes of the two groups. There were no significant differences in age, sex, BMI, hypertension, diabetes, or carotid atheromatous plaque between BPPV patients and controls (all *P* > 0.05), but both C-IMT and vertebral artery stenosis were significantly different between the two groups (both *P* < 0.05). Serum levels of inflammatory factors in the two groups are summarized in Table 2. There was no significant difference in TNF-α and PG-E2 levels between the two groups (*P* > 0.05). However, serum levels of IL-1β, sICAM-1, and sVAP-1 were significantly higher in BPPV patients than controls (*P* < 0.001, *P* < 0.05, and *P* < 0.001, respectively).

**Table 1:** Comparison of clinical parameters between the BPPV and control groups.

Clinical parameters	BPPV(*n* = 90)	Non-BPPV (*n* = 90)	*P* value
	M = 28/F = 62	M = 28/F = 62	
Age, years	55.22 ± 14.67	55.17 ± 14.25	NS
BMI, kg/m^2^	23.83 ± 8.28	22.95 ± 5.21	NS
Hypertension, *n* (%)	23 (25.6)	18 (20.0)	NS
Diabetes, *n* (%)	12 (13.3)	7 (7.8)	NS
**C-IMT,** ***n* (%)**			
Normal	45 (50)	55 (61.1)	
Mild	37 (41.1)	32 (35.6)	< 0.001
Severe	8 (8.9)	3 (0.03)	
**Vertebral artery stenosis, *n (%)***			
Yes	22 (24.4)	9 (10.0)	
No	68 (75.6)	81 (90)	0.010
**Atheromatous plaque, *n (%)***			
Yes	26 (28.9)	20 (22.2)	
No	64 (71.1)	70 (77.8)	NS

Normal, ≤ 0.1 cm; mild, 0.1–0.13 cm; and severe ≥ 0.13 cm.

*BMI* body mass index, *BPPV* benign paroxysmal positional vertigo, *C-IMT* carotid intima-media thickness, *NS* not significant.

**Table 2:** Inflammatory factors.

Inflammatory factors (pg/ml)	BPPV (*n* = 90)	Non-BPPV (*n* = 90)	*P* value
	M = 28/F = 62	M = 28/F = 62	
IL-1β	40.08 ± 8.64	21.19 ± 5.67	< 0.001
TNF-α	48.29 ± 12.64	45.78 ± 11.58	0.168
PG-E2	60.67 ± 11.58	57.64 ± 20.66	0.226
sICAM-1	274.08 ± 102.29	243.59 ± 100.06	0.045
sVAP-1	116.42 ± 22.98	86.13 ± 22.40	< 0.001

*BPPV* benign paroxysmal positional vertigo, *IL-1β* Interleukin-1β, *PG-E2* prostaglandin-E2, *sICAM-1* soluble intercellular adhesion molecule-1, *sVAP-1* soluble vascular adhesion protein-1, *TNF-α* tumor necrosis factor-alpha.

## Discussion

The results of the present study indicated that serum levels of IL-1β, sICAM-1, and sVAP-1 were significantly higher in BPPV patients than in controls. In addition, C-MIT and carotid vertebral stenosis as determined by imaging analysis were significantly different between BPPV patients and controls. These observations suggest that serum IL-1β, sICAM-1, and sVAP-1 levels may be useful as markers of BPPV, and C-MIT and carotid vertebral stenosis may be useful reference factors for BPPV. In addition, inflammation was shown to play a crucial role in the pathogenesis of BPPV.

Güçlütürk et al.^4^ suggested that IL-1β could dominate the inflammatory response in the inner ear. IL-1β is a proinflammatory cytokine that induces multiple inflammatory responses, including lymphocyte amplification, fibroblast growth, adhesion molecule accumulation, and the production of other inflammatory cytokines^23,24^. Indeed, some studies regarding the pathological mechanisms of inner ear disorders indicated the involvement of IL-1β as an inflammatory regulator in some inner ear diseases, including noise-induced hearing loss^25,26^, autoimmune inner ear disease (AIED)^7–9^, and cytomegalovirus (CMV)-induced hearing loss^27^. Cisplatin ototoxicity test indicated that increased IL-6, IL-1β, and TNF-α levels damaged hair cells of the inner ear^28^. IL-1β induces the expression of matrix metalloproteinase-9 (MMP-9), which cleaves IL-1 receptor type II (IL-1RII) and enhances IL-1β-induced signaling^8^. MMP-9 is a member of the family of zinc-dependent metalloproteinases involved in degradation of the extracellular matrix, and functions downstream of the IL-1β-induced signaling pathway^29^. Particularly, MMP-9 was shown to be overexpressed in patients with AIED^30^. In the plasma, IL-1β may contribute to secretion of other proinflammatory mediators, which induce inflammatory reperfusion and worsen inflammation and oxidative stress through blood recirculation.

ICAM-1 is an immunoglobulin superfamily member expressed by leukocytes and endothelial cells. The soluble form of ICAM-1, sICAM-1, is present in plasma and plays an important role in inflammatory responses. Adhesion molecule contributing leukocytes to adhere and migrate as well as soluble adhesion molecule causing endothelium activation, which result in local inflammatory response, endothelial cell damage and plasma leakage^31,32^. sICAM-1 interacts with integrins on the surface of leukocytes, promoting the adhesion of leukocytes to vessel endothelial cells and migration to surrounding inflammatory tissues through the intercellular space. A continuous inflammatory reaction results in increases in leukocyte and platelet binding, leading to vessel endothelial cell damage and dysfunction^33^. The roles and functions of sICAM-1 in inflammatory diseases have been elucidated, but no previous studies have proposed its involvement in inner ear disease. sICAM-1 has been shown to mediate the passage of cells across the blood–brain barrier, which allows peripheral cytokines to enter the central nervous system (CNS)^34^. This study suggested that sICAM-1 enters the inner ear tissue across the blood–labyrinthine barrier and induces an inflammatory response, and elevated plasma sVCAM-1 level results in vasoconstriction and leads to ischemia of the inner ear. Our results suggested a potential role of sICAM-1 in the etiopathogenesis of BPPV.

sVAP-1 has been shown to directly or indirectly result in alterations in the levels of the main factors involved in the pathogenesis of a number of human diseases, including atherosclerosis, obesity, diabetes, stoke, ophthalmological diseases, inflammatory bowel disease, and liver disease^35^. VAP-1 is a membrane-bound adhesion molecule that is involved in inflammation through induction of the migration of leukocytes into inflamed issue^36,37^. sVAP-1 is the soluble form that is present in the circulation^14^, and acts as SSAO, which induces vascular endothelium injury via the generation of vascular damage factors, direct oxidative damage, amyloid deposition, and elevated blood pressure^38^. An animal study showed that VAP-1 inhibitor reduced ICAM-1 levels^39^. Thus, there is a functional relation between VAP-1 and ICAM-1. Although there have been no previous reports regarding the role of sVAP-1 expression in the inner ear, our study suggested that it may participate either directly or indirectly in the pathophysiological mechanism of BPPV by causing vascular dysfunction in the inner ear. The results of the present study suggested that inflammation plays a pivotal role in the pathogenesis of BPPV, in which the interactions of sVAP-1, sICAM-1, and IL-1β play important roles.

**Figure 1:**
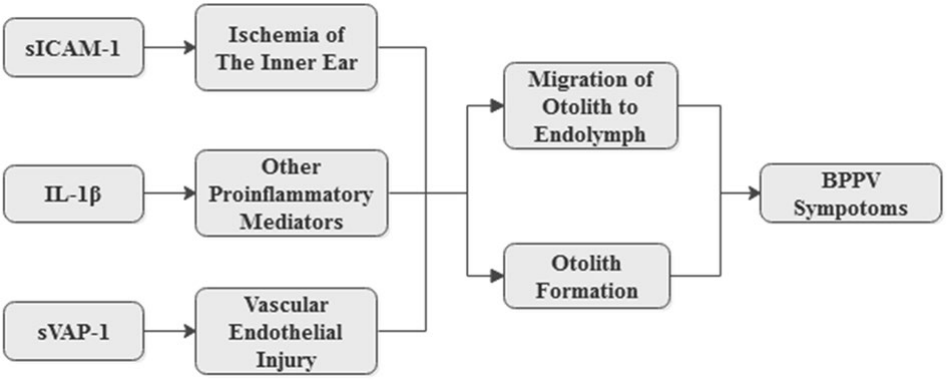
Possible mechanisms triggering the inflammatory response in the inner ear.

BPPV, benign paroxysmal positional vertigo; IL-1β, interleukin-1β; sICAM-1, soluble intercellular adhesion molecule-1; sVAP-1, soluble vascular adhesion protein-1.

However, our results indicated no significant relations of TNF-α and PG-E2 and with the pathogenesis of BPPV, in contrast to the literature, which may have been due to the timing of blood sample collection during the course of the disease. Although we did not perform further verification, previous studies indicated that IL-1β, TNF-α, and PG-E2 played synergistic or complementary roles in contributing to the inflammatory reaction^11,12^. The possible signaling pathways involving sVAP-1, sICAM-1, and IL-1β are illustrated in Fig. 1.

The results of the present study suggested that C-IMT and vertebral artery stenosis may affect the microcirculation of the otolithic organs. Ultrasonographic measurement of C-IMT is a noninvasive imaging technique to evaluate subclinical vascular disease^40^. Although many studies have shown that C-IMT has important diagnostic significance for cardiovascular diseases^17,40,41^, there have been no previous reports of a correlation between C-IMT and circulation of the inner ear. Vertebral artery stenosis leads to decreased blood flow in the anterior inferior cerebellar artery territory, which is involved in the circulation of the vestibular organs, resulting in inner ear dysfunction^42^. Our observations showed that C-IMT and vertebral artery stenosis can be used as reference factors for the diagnosis of BPPV, but further studies are required to verify the correlation between C-IMT and vertebral artery stenosis in BPPV.

## Conclusion

This study suggested that IL-1β, sICAM-1, and sVAP-1 may play roles in the pathogenesis of BPPV, and that C-MIT and carotid vertebral stenosis can be used as reference factors for the diagnosis of BPPV. Whether these factors have cause and effect relationships in BPPV remains unclear, so further studies are needed to evaluate and verify these findings.

## Data Availability

The data supporting the findings of this study are available from the corresponding author upon reasonable request.
