# Author Correction: Carotid imaging changes and serum IL-1β, sICAM-1, and sVAP-1 levels in benign paroxysmal positional vertigo

**DOI:** 10.1038/s41598-021-86988-4

**Published:** 2021-03-25

**Authors:** Xiaoxu Chen, Huimin Feng, Hongjin Liu, Xianrong Xu, Jianchang Wang, Zhanguo Jin

**Affiliations:** 1 Aerospace Balance Medical Center, Chinese PLA Air Force Medical Center, Beijing, 100142 China; 2 People’s Liberation Army Troops of 95935 Unit, Changchun, 130000 China; 3 Hebei North University, Zhangjiakou, 075000 China; 4 Department of Medical Identification, Chinese PLA Air Force Medical Center, Beijing, 100142 China; 5 Chinese PLA Air Force Medical Center, Beijing, 100142 China; 6 Aviation Physiology Identification and Training Laboratory, Chinese PLA Air Force Medical Center, Beijing, 100142 China

Correction to: *Scientific Reports* 10.1038/s41598-020-78516-7, published online 09 December 2020

In the original version of this Article, Jianchang Wang was incorrectly listed as the corresponding author. The correct corresponding author for this Article is Zhanguo Jin. Correspondence and request for materials should be addressed to ccjzg@qq.com. This error has now been corrected in the HTML and PDF versions of the Article.

